# Gleanings in Science and Medicine

**Published:** 1893-09-23

**Authors:** 


### GLEANINGS IN SCIENCE AND MEDICINE.

The Bristol Town Council is doing a good work in the cause of education; £5,200 a year is now being devoted to the furtherance of technical instruction. This is chiefly effected through aiding colleges and schools to give organised courses of lectures in science and art, as also for throwing open scholarships for the same, amongst which subjects chemistry will receive prominent attention.

Although the donor of a gift has the right to attach whatsoever conditions he may see fit with regard to it, yet it must be admitted that Lord Bute, in endowing the Welsh School of Medicine at Cardiff, has taken a full license in this respect. The proviso, on which he makes his contribution dependent, demands that vivisection is not practised within the boundaries of the institution. From a professional standpoint it appears almost beyond the province of a layman to decide in any arbitrary terms what should, or should not, be practised in a medical college.

A great effort has been made lately to bring again into fashion the "grape cure," once so much in vogue on the Continent. The promulgators of this remedy certainly preach a pleasant gospel, and one which few would object to follow, but whilst not disputing the valuable physiological effect of the fruit which is duly assigned thereto, it must be remembered that precisely the same results can be attained at considerably less a cost than this "cure" and its régime would entail. Grapes must, with most nations, anyhow, continue to be the rich man's luxury and not the poor man's food, and the cure connected with them, pleasant and beneficial as it is, barely comes within the province of practical medicine.

It has been calculated by the Metropolitan Drinking Fountains and Cattle Trough Associations that the number of persons who have benefited through the operations of the society and thus quenched their thirst at six fountains during 24 hours reaches the high figure of 25,356, whilst the total number of horses, at the same method of calculation, is 13,401. The services of the Drinking Fountains Association during the recent hot weather have been an incalculable boon to wayfarers, man and beast, and a society which advertises itself so little and confers benefits such as this deserves not only praise from all quarters, but also gratitude expressed in a more substantial form.

The inmates of a certain workhouse in the metropolitan area have found a friend in the Local Government Board inspector. It appears that by the rules of this infirmary (which shall be nameless) the inmates were called at the early matutinal hour of five a.m. in order to get their beds made! At the happiest of times this order would make a day very long; to aged and infirm persons it would still less enhance the joys of living through it. Another tale out of school, regarding the same institution, tells that the beverage generally known as tea in the establishment was little else but coloured water, the allowance for this so-called "tea" being at the generous rate of one ounce of leaves to a gallon of water. In pointing out this sorry state of things to the Board the inspector has proved himself a good friend to the pauper inmates of this particular workhouse.

Faith-curers have again been about their mysterious work, and another life has been sacrificed at their altar, the latest victim in this case being an American child, who was suffering from an attack of diphtheria. A faith-cure preacher was called in, no medical aid was obtained, and while under the care of the preacher the child died. The story is unhappily only too common a one, and does not stand alone as an isolated example of the effects of credence in this particular line of cure; but in this case an investigation has been instituted to determine the responsibility of the parties. This is a most wise and timely step. Faith-cures may or may not be a fact; but, at least, it is certain that their use in the hands of ignorant and unscrupulous persons is doing an ever-increasing amount of harm. In some cases cures may have been effected, but very few are substantiated sufficiently by evidence to bear any careful sifting, and the number of even reputed cases is small indeed when compared with the cases of failure. Faith-cures in various forms have existed contemporaneously with disease, and the belief in their efficacy appears, among the less cultured classes, to be increasing rather than lessening. We await, therefore, with special interest the result of the present investigation connected with this matter, since any trustworthy facts elicited in connection with a subject so obscure must possess a value in excess of those generally obtained by legal proceedings.

To gymnastics, therapeutical as pedagogical, the Swedish nation owes its hardihood, its stature. In a gathering of various nations the Swede stands out prominently as a type of healthy physique—the men tall and broad shouldered, the women healthy and well built. The pages of a weekly contemporary have dealt with this subject in a more exhaustive form, and point out the significance of calisthenic exercise in the development of a race. Recent statistics indeed show that girls who have practised gymnastics are taller than those in the same school who have not gone through this form of exercise, and the same rule holds good with both sexes. In Scandanavia the "higher education" does not alone confine itself to the mind, but embraces the empire of the body also; to what advantage is readily exemplified in the traditionally hardy Norseman himself. In the Swedish student's life mental training goes hand in hand with the physical, whilst as definite a course is also prescribed for the soldier and the sailor, a course which alone is terminated through an examination being satisfactorily passed. The Central Institute at Stockholm, which provides for this particular department of the national training, is under Government management, and embraces a gymnasium in its two-fold character of therapeutic and pedagogic. The medical department provides practical treatment for patients, and so great is the demand for massage that when human hands cannot keep pace with it steam is brought into play, and acts in an ingenious manner the manual part. Through these methods it is that the Swede braces himself up in his muscular strength, choosing rather the exterior to the interior means of attaining to physical well-being. For this reason it is that apothecaries gain but a precarious livelihood in the Swedish capital, and chemists' shops are conspicuous by their absence.

